# Decision-Making and Abuse, What Relationship in Victims of Violence?

**DOI:** 10.3390/ijerph20105879

**Published:** 2023-05-19

**Authors:** Giulia Lausi, Jessica Burrai, Michela Baldi, Fabio Ferlazzo, Stefano Ferracuti, Anna Maria Giannini, Benedetta Barchielli

**Affiliations:** 1 Department of Psychology, Sapienza University of Rome, Via dei Marsi 78, 00185 Rome, Italy; 2 Department of Human Neuroscience, Sapienza University of Rome, Piazzale Aldo Moro 1, 00185 Rome, Italy; 3 Department of Dynamic and Clinical Psychology, and Health Studies, Sapienza University of Rome, Via degli Apuli 1, 00185 Rome, Italy

**Keywords:** sexual abuse, psychological abuse, intimate partner violence, stay-or-leave, domestic violence, violence against women

## Abstract

Gender-Based violence is a worldwide persisting phenomenon: during their lifetime, 30% of women have experienced sexual and/or physical violence. The literature has investigated for several years the association between abuse and possible psychiatric and psychological consequences which may occur even after many years. The most common consequences involve mood and stress disorders (e.g., depression and PTSD). These disorders seem to have secondary long-term effects, such as decision-making and cognitive function impairments. Therefore, the present literature synthesis aimed to investigate whether and how the decision-making capacities of individuals experiencing violence can change because of abuse. We conducted a thematic synthesis using PRISMA guidelines: through a double-blind procedure, 4599 studies were screened; a total of 46 studies were selected for full-text reading, which was reduced to 13 by excluding papers with a wrong focus. To better understand the results of the thematic synthesis, two main focuses have been identified: “leave or stay decision making” and “multifactorial dimensions of decision making”. Results showed that decision-making is an important process in avoiding secondary victimization.

## 1. Introduction

Gender-based violence (GBV), also known as violence against women (VAW), is a phenomenon persisting worldwide: during their lifetime, 30% of women have experienced sexual and/or physical violence [1,2,3]. GBV includes controlling, coercive, threatening, degrading, and violent behavior, including sexual violence [4], which violates the individual’s integrity, both from a physical and psychological point of view, regardless of its characteristics (e.g., age, race, ethnicity, educational level, marital status) [5,6,7]. There can be multiple forms of GBV during one’s lifetime, such as selective abortion [8,9], abandonment [10,11], or non-consensual sharing of intimate images [12,13]. In most cases, the perpetrator knows the victim and has a familial (domestic violence, DM) or romantic (intimate partner violence, IPV) relationship with her. More specifically, IPV refers to a pattern of physical, sexual, or psychological abuse towards a partner or former partner [14,15]. Thus, it is referred to as an ongoing pattern of violence even after a broken-up relationship.

GBV is associated with numerous risk factors. Studies have shown that women from marginalized groups are more likely to experience violence. For instance, women living in poverty or areas with poor infrastructure and limited resources are more vulnerable to GBV than those from wealthier backgrounds. Similarly, women from ethnic or racial minorities may face additional barriers in accessing services and support [16]. In some cases, cultural beliefs and practices can also contribute to GBV. For example, certain cultures may condone violence against women or view it as a private matter that should not be reported to authorities. Geographical factors can also play a role in GBV. Women who live in socially and geographically isolated areas may be more susceptible to abuse because they have limited access to support services and may be more isolated from their communities [17,18]. Similarly, women living in conflict-affected areas or areas with high levels of crime may be more at risk of violence. Relational factors, such as marital status, intra-partner dependence, intergenerational transmission of trauma, and lack of social support, can also contribute to GBV. Women who are dependent on their partners for financial support or have limited access to social networks may find it more difficult to leave abusive relationships. Intergenerational transmission of trauma can also be a factor, where women who have experienced violence in their families of origin may be more likely to experience it in their own relationships [19,20]. Individual factors, such as the presence of a disability and/or cognitive impairment, as well as drug and alcohol use, can also increase the risk of GBV [21,22]. For example, women with disabilities may face additional barriers to seeking help and accessing support services, while drug and alcohol use can increase the likelihood of violent behavior.

These factors could affect both the likelihood of being at risk of abuse and the chances of breaking out of the cycle of violence [23,24,25]. However, no single factor seems to explain why groups or individuals are at a higher risk of victimization or have a greater propensity towards perpetration [26,27].

It is important to consider that gender-based violence arises as a public health problem and that it can have primary consequences (i.e., closely related to the incidents of violence and generally related to the short term) and secondary consequences (i.e., related to the chronicization of the primary consequences). It must be considered that most research on gender-based violence refers to domestic violence, as this is statistically the most frequent form of abuse.

### 1.1. Health Consequences of GBV

The consequences of gender-based violence could have important effects on the health of victims [28]. Violence brings with it much suffering and constitutes a traumatic experience in the personal history of victims, particularly DV victims [29,30], with different patterns of consequences. Victims of violence seem to be at greater risk of unwanted pregnancies, infections, sexual dysfunctions, and abortion [31]; moreover, in some countries, such as the United States of America, GBV seems to be the primary cause of injury in women [30]. Although most of these findings are based on studies about domestic violence, these consequences are not limited to intra-family violence, but also include gender-based violence; a study conducted in six different European countries [32] found that among women who reported having an unwanted pregnancy, 24.5% were abused during their lifetime and 38.5% were recently abused.

The literature has investigated for several years the association between abuse and possible consequences at the psychiatric and psychological level [33]; these consequences may even occur after many years [34,35,36,37]. More specifically, strong evidence suggests a relationship between abuse and eating disorders, sleep disorders, anxiety disorders, suicide attempts, and somatic symptoms [33]; mostly, victims seem to experience depression and post-traumatic stress disorder (PTSD). Women who have been abused, both in childhood and adulthood, were more likely to suffer from depression (56.2%) than childhood-only abused women (33.9%), suggesting a cumulative impact of violence experience on mental health [38]; moreover, regarding post-traumatic stress disorder, some studies have highlighted how among its possible causes, besides sexual and physical abuse [39] which are the most studied ones, there is also psychological abuse [40] even though is often underestimated. The severity of psychiatric symptoms seems to increase with the severity of the violence [41]; moreover, women who report experiencing IPV over the past year were more likely to report PTSD than women who report experiencing other forms of violence [42]. Women who live in abusive relationships or fear violence are more vulnerable to contracting sexually transmitted diseases, as they have less control over the time or circumstances of sexual intercourse and are also less able to negotiate the use of condoms [43].

Another consequence of GBV and particularly DV, the most immediately obvious, is physical and non-fatal injuries, with the head, neck, and face as the most affected regions, followed by the musculoskeletal and genital regions [44]. Several studies [45,46,47] suggest that adult women who have experienced at least one episode of abuse in their lives experience a wide range of depressive symptoms, including sleep and appetite difficulties, loss of interest in normal activities, and concentration problems. These primary consequences may lead to secondary consequences in the long term that are not directly related to gender-based violence but rather to the chronicization of symptoms.

### 1.2. Secondary Consequences of GBV

The health consequences of abuse may also have long-term secondary outcomes which could be related to the primary ones. The chronicity of mood and depressive disorders resulting from experiencing gender-based violence can also have important consequences on those processes that are not directly related to GBV, such as cognitive impairment. Individuals suffering from depression may experience impairments in the executive [48] and cognitive functions (e.g., attention and memory [49,50]). As we have seen so far, violence has serious consequences on the physical and mental health of its victims. These effects may also occur in the decision-making process of those who suffer from aggression. Unfortunately, little research has attempted to investigate how these consequences may occur and what changes they are capable of producing. Particularly, a recent study [51] found that individuals suffering from Major Depressive Disorder seem to need more time to make decisions and show biased decision-making strategies [52,53]. Moreover, some relationships between the physiological response to stress and functions, such as attention, executive functions, and decision making, have been highlighted [54]. Symptoms’ severity seems to be related to the individual tendency to suppress painful contents and to the mitigated planning strategies (e.g., the tendency to take fewer risks or to spend more time making decisions) [55,56]. Deciding implies a simultaneous evaluation of present stimuli and possible choices [50], which subconsciously involves information reduction while reasoning [57,58,59], and is also affected by moods and emotions in the post-reasoning evaluations and therefore decision-making processes [60,61].

### 1.3. Aim of the Present Work

The present review originates from the lack of a widespread systematic study of the link between the experience of gender-based violence and decision making and aims to fill the gap in the international literature. In particular, the question has been asked regarding whether and how the decision-making abilities of individuals experiencing violence may change as a result of the abuse. To conduct such a review, quantitative and possibly longitudinal research is needed. However, the results of the preliminary literature research have highlighted the challenge of carrying out a systematic review considering the lack of studies whose results could be compared with each other. Following the indications from Thomas & Harden [62], a thematic synthesis has been carried out to provide a first overview of the current state of the research on the topic.

## 2. Materials and Methods

This current review was performed according to the recommendations of the “Preferred Reporting Items for Systematic Reviews and Meta-Analyses” (PRISMA). The study was registered in the “International Prospective Register of Systematic Reviews” (PROSPERO) in July 2020 (CRD42020187457), and the detailed protocol is available upon request.

The bibliographic research was conducted in parallel on 4 databases (PubMed/MEDLINE, PsycINFO, Web of Science, Scopus) from January to May 2020; new research was conducted from September 2022 to November 2022 to retrieve newly published papers. The search strategy included terms related to abuse (including “sexual abuse”, “psychological abuse”, “physical abuse”, and “economic abuse”) and decision making; the search terms have been adapted for use in different bibliographic databases and combined with specific filters, if available; searches included combinations with Boolean operators (i.e., AND, OR). Studies published in English, Italian, French, and Spanish have been included. The reference lists from retrieved studies have been searched to look for studies that might not have been included in the database’s research. Authors of unpublished or unavailable studies were contacted to obtain a copy of their research; not all papers have been obtained. The citations have been exported into Mendeley Desktop software (Version 1.19.4). Studies about decision-making capacity and the presence of any previously experienced abuse, and whose sample consisted of individuals (regardless of gender) from 17 years old and over, have been included. Eligible studies were intended to measure decision-making skills in individuals who have been abused, including qualitative and/or quantitative studies and cross-sectional studies, correlation studies, cohort studies, case-control studies, audits, prospective studies, and trials where other inclusion criteria are met. Studies involving disorders that might affect decision making (e.g., substance abuse) in their sample have been excluded since it would not be possible to assess decision making in gender-based violence without bias due to the already existing relationship between decision making and the disorders.

The studies were selected and screened in a double-blind procedure, following the inclusion/exclusion criteria. In the case of a disagreement among the researchers, first, there was a comparison between them; in the case of a disagreement, a third expert was involved in the final decision.

**Figure 1:**
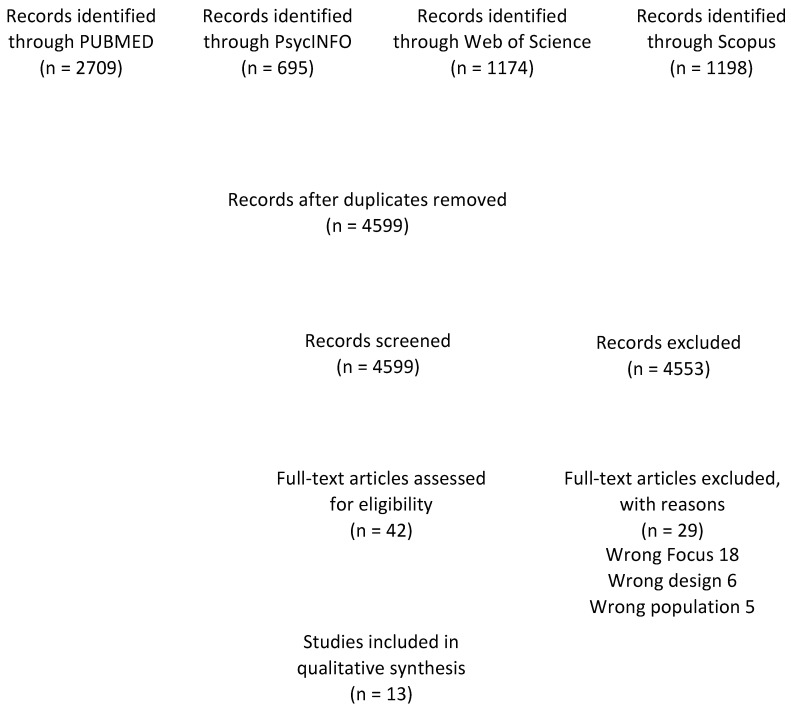
Flow Chart.

The searches identified 5776 primary studies; duplicates between the databases were checked (1177, 20.38% of the total studies). The remaining 4599 papers were screened for titles and abstracts to assess whether the topics were likely to agree with the aim of the review. 46 studies were selected for full-text reading, which was reduced to 13 by excluding papers with a wrong focus (e.g., not including gender-based violence or decision making; n = 18), wrong design (e.g., reports or case studies; n = 6), or wrong population (e.g., children or adolescents; n = 5) (Figure 1).

Materials have been extracted from the selected articles and incorporated into an Excel spreadsheet; the included data were demographic characteristics (e.g., gender, age, ethnicity), study design, and measures for assessing the presence of the abuse and for evaluating decision making (Table 1).

## 3. Results

Most of the studies included in the review are qualitative and involve a total number of 90,106 subjects (males = 57, females = 90,049; non-victims = 119, victims = 89,987). Most of the studies did not investigate the severity of the abuse which was self-reported by the participants; three studies used the Conflict Tactic Scale (CTS) in different versions; one research study used the Sexual Experience Survey (SES). As far as decision making is concerned, both interviews and validated scales have been used. Three studies used interviews, four other studies used semi-structured interviews, two used vignettes, and one analyzed focus group discussions. One study used Real Moral Conflict and Choice Interviews, Need Satisfaction, and Global Satisfaction with Current Relationship, regarding validated scales.

Since decision-making can be measured and evaluated in different ways and from different theoretical assumptions, we identified two thematic approaches to facilitate the interpretation [62],: the first concerns decision-making as a process of choice in remaining within the abusive relationship or moving away from it. The second concerns the multidimensionality of decision-making, which is also characterized by components such as risk perception, help-seeking behavior, and emotional regulation (e.g., fear).

### 3.1. Leave-or-Stay Decision-Making

These studies provide a review of the factors that influence women’s decisions to leave or stay in abusive relationships. This kind of decision-making process is highly affected by the victim’s individual experience: what are her expectations of a possible future with or without the abuser? How does her own social network operate? Does she feel supported by the people around her? The answers to these questions, which are highlighted explicitly in Choice and Lamke [66], are the common thread among all the studies reviewed. It is in this context that the social aspect of domestic and intimate partner violence emerges most prominently. Adjei et al. [63] explored leave or stay as a cultural factor and identified two discursive patterns: (a) stay to protect the family image and (b) remain or leave as the product of negotiation and one’s own agency capacity. Belknap [65] also investigated the first pattern by explicating the decision to leave an abusive relationship as a moral conflict. Here it is necessary to understand the importance of relationships for a woman’s sense of self and the influence of social pressures on her sense of responsibility to maintain an abusive relationship. The second pattern emphasizes the importance of the agency as an important internal resource used by women to deal with the process of leaving [73]. In Patzel [73], the agency was divided into three components: self-education, voice (seen in the women’s self-talk and telling of their stories), and spirit (in the form of faith) were all considered very important in the process of separation from abusers. Johnson [70] explored correlating reasons for battered women’s decisions to return home to their aggressor after being placed in a shelter. Four factors emerged: annual family income, employment status, the severity of the abuse, and the victim’s self-perception. According to Estrellado & Loh [68], several factors contribute to the two different processes of staying and leaving. Intrapersonal and interpersonal factors were associated with the decision to stay, such as personality characteristics, lack of personal resources, absence of social support, presence of children, length of the relationship, and sociocultural factors. Battered women’s decision to leave their abusive partners was associated with factors such as personality characteristics, personal resources, social support, nature of abuse, and spousal factors. Choice and Lamke [66] investigated a conceptual model of the stay/leave decision-making process in an abusive relationship using two factors “Will I be better off?” and “Can I do it?”. The results identify “Will I be better off?” as one superordinate construct defined by relationship satisfaction, quality of alternatives, irretrievable investments, and subjective norm. Instead, the study failed to operationalize and measure the “Can I do it?” factor and couldn’t adequately test its effects in the model (Table 2).

### 3.2. Multifactorial Dimensions of Decision-Making

Research findings show that overall sexual and physical violence committed by a partner reduces the decision-making abilities of victims; individual characteristics are linked to IPV experience, for instance, older age, educational level, the age at marriage, having witnessed childhood violence, and the decision-making autonomy; besides, family characteristics seem to play a role in IPV, such as educational level of the husband or the presence of alcohol drinking in the husband [64]. Considering decision making as a multifactorial dimension, different variables have been taken into consideration from the studies. For instance, the attitudes toward gender equality led to higher levels of decision-making skills [75], functioning as a partial mediator between the decision-making capacity and episodes of psychological, physical, and sexual violence. Moreover, when asked to classify high-risk situations, victimization history seems to be a predictor of lower-risk classification; this classification differs also according to the severity of victimization (i.e., the more severe the victimization experience, the fewer high-risk situations were classified); another variable that seems to play a role in risk-assessment of vignettes and decision-making capacity in victimization history is rape myth acceptance which moderates the explicit judgments about risks [74].

An important factor of decision-making processes in dealing with abusive situations is the centrality of motherhood in women’s lives; motherhood is the primary responsibility of many battered women and is the yardstick for their choices. The impact that leaving their father may have on their children, the economic conditions, and the cultural values that are placed in the first place regarding their well-being and, when the abusive relationship is interrupted, this is for the sake of the children [71]. Similar results have been found in a study by Frisch & MacKenzie [69]: when asked about why they remained in their abusive relationship, answers ranged from the absence of a safe place to go to the feeling that their children needed the support from their father or the pressure from their family of origin.

When compared with women who have not experienced violence, battered women consistently show a high desire for alternatives to their relationship, but also greater insight into their strategies than the non-abused sample; this emerged in a study from McDonough [72], where differences among the group were investigated on relationship commitment through the MDRCI and vignettes of hypothetical family altercations. Within this second theme, more cognitive aspects emerge (although equally related to the social context), linked not only to individual experience but also to the general functioning of the individual. Unfortunately, despite the attempt at a more quantitative perspective in these studies (including through the use of validated scales), a unified approach to the topic is still lacking (Table 2).

## 4. Discussion

The examined studies show how decision making within family abuse situations is a fundamental component used to get out of the cycle of domestic violence [23]. Deciding involves, in abused women, a variety of processes requiring a high cognitive and emotional load. While women are asked for the ability to exit an abusive situation, they are also asked to think about the family image, to fulfil their responsibilities as mothers, thus creating a first moral conflict in the victim’s decision making [63,66,68,71,73]: in this phase of exiting from the abusive situation, the presence of children, the family’s annual income, the work situation, the severity of experienced abuse, and self-esteem also play a role, which in turn become factors affecting the likelihood of returning to the abusive situation. Notably, when victims of abuse are mothers, their role as caregivers is placed ahead of their well-being: the woman finds herself having to consider whether it is appropriate for her children to stop seeing their father, or the economic impact that separation might have on their children’s lives [69,71]. People’s decision-making ability, identity, mental and physical health, anxiety levels, and social and economic development are all affected by violence. Individuals who feel safe prioritize values that are linked to self-expression and quality of life, in contrast to those who have experienced violence [76]. Violence against women can limit their ability to make decisions in two ways. Firstly, it shapes the victim’s experience, influencing their beliefs and molding them to fit societal and familial roles. This indirectly impacts their decision-making ability. Secondly, violence can have a direct impact on a woman’s ability to make decisions. Physical, sexual, and psychological abuse can impair their capacity to make choices, while economic violence can increase it. Women may be more inclined to seek ways to maintain economic stability in the household rather than resorting to direct decision making when it comes to economic violence [77].

The impact of egalitarian attitudes on victims’ decision making would appear to be important: women who have a less traditional and more egalitarian vision of their role in society have a stronger agency, a greater sense of empowerment, and perceive themselves as free to decide to defend their rights and improve their well-being. In Rodriguez and colleagues’ study [75], this relationship was also thoroughly investigated according to the type of violence reported by the victims.

## 5. Conclusions

This review revealed how complex it is to be able to uniquely define decision making within such a large and multifaceted social phenomenon. Although the number of studies included in the qualitative synthesis is small, the evaluation of the studies through the eligibility criteria provided insight into the complexity of investigating decision making within gender-based violence. Therefore, it must be considered that the studies identified in the present review are extremely varied, also because of the multidimensional aspects of gender-based violence and decision making. It emerges from the considerations made through the studies that the victim’s perception of violence has a bearing on their subsequent decisions. For example, if a topic generates anger in the partner, the victim may feel that she has no control over that topic. Perceived control influences motivation to act [78] and behavior, also based on the simultaneous behavior of another individual [79,80]. When looking at relational decision-making processes within this theory, intention, attitude, and subjective norms play a role in the resulting behavior. Thus, when one partner gains control over the other through the use of force, the woman’s perceived control over her partner may influence the development of traditional modes of behavior. According to Allport [81], these attitudes take the form of a mental and neural state of readiness, which is organized through experience, exerting, in a more or less direct way, an influence on the ways in which the individual responds to the various situations in which he or she acts. Furthermore, such attitudes are charged with emotions that guide behavior and consist of beliefs and feelings that determine people’s intentions [82]. Therefore, attitudes support and give meaning to individuals’ behavior [83].

Unfortunately, the literature on the association between decision-making processes and violence is still limited and investigated with different techniques. This raises an important limitation to the present literature review as it does not make it possible to uniquely compare results. In fact, comparison between qualitative and quantitative data and with different approaches poses a methodological limitation that must be considered. At the same time, however, it provides a perspective toward future research that could be developed. Indeed, future studies could draw on the theoretical background already present in the literature to investigate the effects of violence on cognitive processes, especially because of their importance in avoiding secondary victimization. In addition, understanding the many aspects and factors that influence decision making within intimate partner violence dynamics will allow the identification of gaps in the literature in order to define the different areas of intervention. Based on the current thematic synthesis and the growing research interest in gender-based violence, it is important to consider possible future perspectives, such as the expansion of current knowledge through literature reviews that include specific variables or factors identified in the present qualitative synthesis. Additionally, it has emerged that experimental research can also be expanded within the sample of violence victims, evaluating not only cognitive variables but also factors such as the age at which victimization occurred and the period of time elapsed between the abuse and seeking help. Furthermore, child and adolescent populations could also be included in studies to allow for specific comparisons of any age-related differences in adulthood. From the current state of the literature, longitudinal studies could also be developed to track changes in decision making over time, and to identify which factors influence behavioral and decision-making trends. Finally, the present literature analysis could enable new intervention studies to develop evidence-based support programs that consider decision-making processes.

## Figures and Tables

**Table 1:** Studies on Decision-Making in Gender-Based Violence Victims.

First Author (Year)	Study Focus	Study Design	Sample					Measures	
Country			Controls (C)	Abused (A)	Ethnicity	Gender	Age	Abuse	Decision Making
[63]	What contributes in stay/leave decision making	Qualitative Study	NA	16	African	Female	24–60 y.o.	FGDs; Interview	
Denmark									
[64]	Factors associated with IPV in married women	Cross-sectional Study	NA	3.373	Asian	Female	M = 31.09 SD = 8.26	CTS; ad-hoc items indicators of domestic violence	ad-hoc items for decisions and choices
Korea									
[65]	Decision making in battered women (stay/leave; threat; resistance)	Qualitative Study	NA	18	different ethnicities	Female	35–51 y.o.	Self- reported	RLMCCI; Fable Method (semi-structured interview)
USA									
[66]	What contributes to stay/leave decision making	Explorative Study	NA	126	NS	Female (70); Male (56)	adults	Self- reported; CTS-N	RAS; NSCR; GSCR; NSAR; GQS; EFAPI; Investment Scale; Ad-hoc Measure of investment items; RES; SNA; Self-Mastery Scale; PBC; DAdjS; TSERR
USA									
[67]	Decision-making model for help seeking	Qualitative Study	NA	14	different ethnicities	Female (13); Male (1)	19–25 y.o. M = 21.29	Self-reported Unwanted Sexual Experiences	Semi-structured interview
USA									
[68]	Stay/leave decision making in battered women	Qualitative Study	NA	40	Filipino	Female	M = 39.78 SD = 9.79	Self- reported	Interviews
Australia									
[69]	Differences between chronically and formerly battered women	Nonrandomized Controlled Study	NA	46 (23CA; 23 FA)	NS	Female	M = 32.66 SD = 8.18	Screening interview	ATWS–SF; Self-Esteem Inventory; Internal-Powerful Other-Chance Scale-Chance Subscale; DAS; Social Avoidance and Distress Scale; Assertion Inventory; SAFA
USA									
[70]	What contributes to stay/leave decision making	Qualitative Study	NA	412	different ethnicities	Female	adults	Semi-structured interviews	
USA									
[71]	Decision-making in battered mothers	Qualitative Study	NA	17	Latino	Female	19–53 y.o.	Interviews	
USA									
[72]	Decision-making in battered and non-battered women	Nonrandomized Controlled Study	30	28	different ethnicities	Female	M = 31.07(C) M = 26.7 (A)	CTS-R	MDRCI; vignettes
USA									
[73]	What contributes to stay/leave decision making	Qualitative Study	NA	10	NS	Female	35–58 y.o.	Semi-structured Interview	
USA									
[74]	Rape Myth Acceptance and Risk-Judgments in abused and non-abused women	Correlational Study	89	105	different ethnicities	Female	18–24 y.o.	SES	RMAS; Vignettes
USA									
[75]	Decision-Making and Gender-Equality in battered and non-battered women	Correlational Study	NA	85.782	Mexican	Female	adults	Survey	
Mexico									

Notes: CA = Chronically Abused; FA = Formerly Abused

**Table 2:** Results of the studies.

First Author (Year)	Study Focus	Results
Country		
[63]	What contributes to stay/leave decision making	- Staying in an abusive relationship to preserve family image - Staying or leaving as the result of a negotiation within familial and cultural values
Denmark		
[64]	Factors associated with IPV in married women	- Lack of formal education, witnessing violence in childhood, lack of decision-making autonomy, and an alcoholic husband are the main risk factors for IPV
Korea		
[65]	Decision making in battered women (stay/leave; threat; resistance)	- Protecting self and children may help in leaving an abusive relationship - Love and sympathy for the abuser are the reasons to stay - The fear of losing the relationship with children/friends might be a reason to stay - Preserving the relationship between father and child is one of the reasons to stay
USA		
[66]	What contributes to stay/leave decision making	- The decision-making process is affected by internal carachteristics (personal resources, intentions, orientation toward the future, subjective norms) and external factors (alternatives, structural resources)
USA		
[67]	Decision-making model for help seeking	- The helpseeking process after a sexual assault implies decisions about informal resources, seeking help, coping.
USA		
[68]	Stay/leave decision making in battered women	- Staying or leaving an abusive relationship is affected by intrapersonal factors (i.e., personality characteristics, personal resources), interpersonal factors (i.e., social support, presence of children, characteristic of the abuse, sociocultural factors)
Australia		
[69]	Differences between chronically and formerly battered women	- Chronically abused woman have more traditional attitudes about women’s role, lower self-esteem, less educated than formerly abused - There are no differences in dysfunctional attitudes, social anxiety, religious activity
USA		
[70]	What contributes to stay/leave decision making	- Economic, situational and psychological characteristics were significantly related to the abusive relationship
USA		
[71]	Decision-making in battered mothers	- Being a mother is the main responsibility that weights the decisions: staying or leaving for the sake of the children
USA		
[72]	Decision-making in battered and non-battered women	- Battered women reoported fewer rewards, greater costs and greater desire for alternatives in the relationships than non-battered woman
USA		
[73]	What contributes to stay/leave decision making	- Leaving process follows some common themes: turning points, realization, reframing, agency, self efficacy
USA		
[74]	Rape Myth Acceptance and Risk-Judgments in abused and non-abused women	- Women with more severe victimization history perceived fewer high-risk situations
USA		
[75]	Decision-Making and Gender-Equality in battered and non-battered women	- Egalitarian attitudes and decision-making capacity affect the personal well-being (e.g., a less traditional view of the gender roles is related to the likelihood to defend the rights) - Physical sexual and psychological violence constrains women’s capacity to make decisions
Mexico		

## Data Availability

Data sharing is not applicable.

## References

[1] Devries K.M., Mak J.Y.T., García-Moreno C., Petzold M., Child J.C., Falder G., Lim S., Bacchus L.J., Engell R.E., Rosenfeld L. (2013). The Global Prevalence of Intimate Partner Violence Against Women. Science.

[2] World Health Organization (2020). Global and regional estimates of domestic violence, prevalence and health effects of intimate partner violence and non-partner sexual violence. Lond. Sch. Hyg. Trop. Med..

[3] World Health Organization (2013). Global and Regional Estimates of Violence against Women, Prevalence and Health Effects of Intimate Partner Violence and Non-Partner Sexual Violence.

[4] Council of Europe (2011). Convention on Preventing and Combating Violence against Women and Domestic Violence. Counc. Eur. Treaty Ser..

[5] Alhabib S., Nur U., Jones R. (2009). Domestic Violence Against Women: Systematic Review of Prevalence Studies. J. Fam. Violence.

[6] Campbell J.C. (1995). Adult response to violence. Violence Plague Our Land.

[7] Joachim J. (1999). Shaping the human rights agenda, the case of violence against women. Gend. Polit. Glob. Gov..

[8] Abrejo F.G., Shaikh B.T., Rizvi N. (2009). ‘And they kill me, only because I am a girl’… a review of sex-selective abortions in South Asia. Eur. J. Contracept. Reprod. Health Care..

[9] Purewal N. (2018). Sex Selective Abortion, Neoliberal Patriarchy and Structural Violence in India. Fem. Rev..

[10] McCloskey L.A. (2016). Focus, Sex and gender health, The effects of gender-based violence on women’s unwanted pregnancy and abortion. Yale J. Biol. Med..

[11] Pathak S.J. (2015). Domestic Violence-An Insight into Incest. Nirma ULJ HeinOnline.

[12] Henry N., Powell A. (2014). Beyond the ‘sext’: Technology-facilitated sexual violence and harassment against adult women. Aust. New Zealand J. Criminol..

[13] Cricenti C., Pizzo A., Quaglieri A., Mari E., Cordellieri P., Bonucchi C., Torretta P., Giannini A.M., Lausi G. (2022). Did They Deserve It? Adolescents’ Perception of Online Harassment in a Real-Case Scenario. Int. J. Environ. Res. Public Heal..

[14] Miller E., McCaw B. (2019). Intimate partner violence. N. Engl. J. Med..

[15] Zara G., Veggi S., Gino S. (2020). Intimate Partner Violence, la tipologia della relazione e l’intimità affettiva nelle dinamiche interpersonali violente. Ital. Psicol. Soc..

[16] Hotaling G.T., Sugarman D.B. (1986). An analysis of risk markers in husband to wife violence: The current state of knowledge. Violence Vict..

[17] Lausi G., Pizzo A., Cricenti C., Baldi M., Desiderio R., Giannini A.M., Mari E. (2021). Intimate Partner Violence during the COVID-19 Pandemic: A Review of the Phenomenon from Victims’ and Help Professionals’ Perspectives. Int. J. Environ. Res. Public Health.

[18] Barchielli B., Baldi M., Paoli E., Roma P., Ferracuti S., Napoli C., Giannini A.M., Lausi G. (2021). When “Stay at Home” Can Be Dangerous: Data on Domestic Violence in Italy during COVID-19 Lockdown. Int. J. Environ. Res. Public Health.

[19] Franklin C.A., Kercher G.A. (2012). The Intergenerational Transmission of Intimate Partner Violence: Differentiating Correlates in a Random Community Sample. J. Fam. Violence.

[20] McKinney C.M., Caetano R., Ramisetty-Mikler S., Nelson S. (2009). Childhood Family Violence and Perpetration and Victimization of Intimate Partner Violence: Findings from a National Population-Based Study of Couples. Ann. Epidemiol..

[21] Fals-Stewart W., Golden J., Schumacher J.A. (2003). Intimate partner violence and substance use: A longitudinal day-to-day examination. Addict. Behav..

[22] Stith S.M., Smith D.B., Penn C.E., Ward D.B., Tritt D. (2004). Intimate partner physical abuse perpetration and victimization risk factors: A meta-analytic review. Aggress. Violent Behav..

[23] Eriksson L., Mazerolle P. (2014). A Cycle of Violence? Examining Family-of-Origin Violence, Attitudes, and Intimate Partner Violence Perpetration. J. Interpers. Violence.

[24] Richards T.N., Tomsich E., Gover A.R., Jennings W.G. (2016). The Cycle of Violence Revisited: Distinguishing Intimate Partner Violence Offenders Only, Victims Only, and Victim-Offenders. Violence Vict..

[25] Gómez A.M. (2010). Testing the Cycle of Violence Hypothesis: Child Abuse and Adolescent Dating Violence as Predictors of Intimate Partner Violence in Young Adulthood. Youth Soc..

[26] Hollomotz A. (2009). Beyond ‘Vulnerability’: An Ecological Model Approach to Conceptualizing Risk of Sexual Violence against People with Learning Difficulties. Br. J. Soc. Work..

[27] Thompson N. (2020). Anti-Discriminatory Practice, Equality, Diversity and Social Justice.

[28] Lutgendorf M.A. (2019). Intimate Partner Violence and Women’s Health. Obstetrics & Gynecology.

[29] Lawrence E., Orengo-Aguayo R., Langer A., Brock R.L. (2012). The Impact and Consequences of Partner Abuse on Partners. Partn. Abus..

[30] Tjaden P., Thoennes N. (2000). Extent, Nature, and Consequences of Intimate Partner Violence.

[31] Pallitto C.C., García-Moreno C., Jansen H.A.F.M., Heise L., Ellsberg M., Watts C. (2013). Intimate partner violence, abortion, and unintended pregnancy, results from the WHO Multi-country Study on Women’s Health and Domestic Violence. Int. J. Gynecol. Obstet..

[32] Lukasse M., Laanpere M., Karro H., Kristjansdottir H., Schroll A.-M., Van Parys A.-S. (2015). Pregnancy intendedness and the association with physical, sexual and emotional abuse – a European multi-country cross-sectional study. BMC Pregnancy Childbirth.

[33] Chen L.P., Murad M.H., Paras M.L., Colbenson K.M., Sattler A.L., Goranson E.N. (2010). Sexual Abuse and Lifetime Diagnosis of Psychiatric Disorders, Systematic Review and Meta-analysis. Mayo Clin. Proc..

[34] Flores R.J., Campo-Arias A., Stimpson J.P., Chalela C.M., Reyes-Ortiz C.A. (2018). The Association Between Past Sexual Abuse and Depression in Older Adults from Colombia. J. Geriatr. Psychiatry Neurol..

[35] E MacGregor K., Villalta L., Clarke V., Viner R., Kramer T., Khadr S.N. (2019). A systematic review of short and medium-term mental health outcomes in young people following sexual assault. J. Child Adolesc. Ment. Heal..

[36] Infurna M.R., Reichl C., Parzer P., Schimmenti A., Bifulco A., Kaess M. (2016). Associations between depression and specific childhood experiences of abuse and neglect: A meta-analysis. J. Affect. Disord..

[37] Wielaard I., Hoyer M., Rhebergen D., Stek M.L., Comijs H.C. (2018). Childhood abuse and late-life depression, Mediating effects of psychosocial factors for early-and late-onset depression. Int. J. Geriatr. Psychiatry..

[38] Ouellet-Morin I., Fisher H.l., York-Smith M., Fincham-Campbell S., Moffitt T., Arseneault L. (2015). Intimate Partner Violence And New-Onset Depression, A Longitudinal Study Of Women’s Childhood And Adult Histories Of Abuse. Depress. Anxiety.

[39] Javidi H., Yadollahie M. (2012). Post-traumatic stress disorder. Int. J. Occup. Env. Med..

[40] Começanha R., Basto-Pereira M., Maia Â. (2017). Clinically speaking, psychological abuse matters. Compr. Psychiatry.

[41] Ferrari G., Agnew-Davies R., Bailey J., Howard L., Howarth E., Peters T., Sardinha L., Feder G. (2016). Domestic violence and mental health: A cross-sectional survey of women seeking help from domestic violence support services. Glob. Heal. Action.

[42] Gupta J., Falb K.L., Carliner H., Hossain M., Kpebo D., Annan J. (2014). Associations between Exposure to Intimate Partner Violence, Armed Conflict, and Probable PTSD among Women in Rural Côte d’Ivoire. PLOS ONE.

[43] Wingood G.M., DiClemente R.J. (1997). The effects of an abusive primary partner on the condom use and sexual negotiation practices of African American women. Am. J. Public Heal..

[44] Sheridan D.J., Nash K.R. (2007). Acute Injury Patterns of Intimate Partner Violence Victims. Trauma, Violence, Abus..

[45] Frank E., Stewart B.D. (1984). Depressive symptoms in rape victims: A revisit. J. Affect. Disord..

[46] Frank E., Turner S.M., Duffy B. (1979). Depressive symptoms in rape victims. J. Affect. Disord..

[47] Burnam M.A., Stein J.A., Golding J.M., Siegel J.M., Sorenson S.B., Forsythe A.B. (1988). Sexual assault and mental disorders in a community population. J. Consult. Clin. Psychol..

[48] Snyder H.R. (2013). Major depressive disorder is associated with broad impairments on neuropsychological measures of executive function: A meta-analysis and review. Psychol. Bull..

[49] Biringer E., Mykletun A., Sundet K., Kroken R., Stordal K.I., Lund A. (2007). A longitudinal analysis of neurocognitive function in unipolar depression. J. Clin. Exp. Neuropsychol..

[50] Aupperle R.L., Melrose A.J., Stein M.B., Paulus M.P. (2012). Executive function and PTSD: Disengaging from trauma. Neuropharmacology.

[51] Lawlor V.M., Webb C.A., Wiecki T.V., Frank M.J., Trivedi M., Pizzagalli D.A. (2020). Dissecting the impact of depression on decision-making. Psychol. Med..

[52] Adolphs R., Tranel D., Bechara A., Damasio H., Damasio A.R. (1996). Neuropsychological Approaches to Reasoning and Decision-Making. Neurobiology of Decision-Making.

[53] Bechara A., Damasio H., Tranel D., Damasio A.R. (1997). Deciding Advantageously Before Knowing the Advantageous Strategy. Science.

[54] Lebois L.A., Wolff J.D., Ressler K.J. (2016). Neuroimaging genetic approaches to posttraumatic stress disorder. Exp. Neurol..

[55] Montague P.R., Dolan R.J., Friston K.J., Dayan P. (2012). Computational psychiatry. Trends Cogn. Sci..

[56] Huys Q.J.M., Eshel N., O’Nions E., Sheridan L., Dayan P., Roiser J.P. (2012). Bonsai Trees in Your Head: How the Pavlovian System Sculpts Goal-Directed Choices by Pruning Decision Trees. PLOS Comput. Biol..

[57] Kahneman D., Frederick S. (2002). Representativeness Revisited: Attribute Substitution in Intuitive Judgment. Heuristics and Biases.

[58] Shah A.K., Oppenheimer D.M. (2008). Heuristics made easy, An effort-reduction framework. Psychol Bull..

[59] Evans J.S.B.T., Stanovich K.E. (2013). Dual-Process Theories of Higher Cognition. Perspect. Psychol. Sci..

[60] Bower G.H. (1981). Mood and memory. Am. Psychol..

[61] Schwarz N., Clore G.L. (1996). Feelings and phenomenal experiences. Soc. Psychol. Handb. Basic Princ..

[62] Thomas J., Harden A. (2008). Methods for the thematic synthesis of qualitative research in systematic reviews. BMC Med Res. Methodol..

[63] Adjei S.B. (2017). Entrapment of Victims of Spousal Abuse in Ghana, A Discursive Analysis of Family Identity and Agency of Battered Women. J. Interpers. Violence.

[64] Atteraya M.S., Gnawali S., Song I.H. (2014). Factors Associated with Intimate Partner Violence Against Married Women in Nepal. J. Interpers. Violence.

[65] Belknap R.A. (1999). Why did she do that? Issues of moral conflict in battered women’s decision making. Issues Ment. Heal. Nurs..

[66] Choice P., Lamke L.K. (1999). Stay/leave decision-making processes in abusive dating relationships. Pers. Relationships.

[67] DeLoveh H.L.M., Cattaneo L.B. (2017). Deciding Where to Turn: A Qualitative Investigation of College Students’ Helpseeking Decisions After Sexual Assault. Am. J. Community Psychol..

[68] Estrellado A.F., Loh J.I. (2013). Factors Associated with Battered Filipino Women’s Decision to Stay in or Leave an Abusive Relationship. J. Interpers. Violence.

[69] Frisch M.B., MacKenzie C.J. (1991). A comparison of formerly and chronically battered women on cognitive and situational dimensions. Psychotherapy.

[70] Johnson I.M. (1992). Psychological Correlates of The Decision-Making Process Of Battered Women. Fam. Soc. J. Contemp. Hum. Serv..

[71] Kelly U.A. (2009). “I’m a mother first”: The influence of mothering in the decision-making processes of battered immigrant Latino women. Res. Nurs. Health.

[72] McDonough T.A. (2010). A Policy Capturing Investigation of Battered Women’s Decisions to Stay in Violent Relationships. Violence Vict..

[73] Patzel B. (2000). Women’s Use of Resources In Leaving Abusive Relationships, A Naturalistic Inquiry. Issues Ment Health Nurs..

[74] Yeater E.A., Treat T.A., Viken R.J., McFall R.M. (2010). Cognitive processes underlying women’s risk judgments: Associations with sexual victimization history and rape myth acceptance. J. Consult. Clin. Psychol..

[75] Rodriguez J.O., Palencia E.P., Lagunas E.A. (2017). The Effect of Different Forms of Violence on Women’s Attitudes Toward Gender Equality and Decision-Making Capacity. Affilia.

[76] Wright J.D., Inglehart R. (1991). Culture Shift in Advanced Industrial Society. Contemp. Sociol. A J. Rev..

[77] Turshen M., Holcomb B. (1993). Women’s lives and public policy: The international experience.

[78] Ajzen I. (1991). The theory of planned behavior. Organ. Behav. Hum. Decis. Process.

[79] Ajzen I., Fishbein M. (1980). Understanding Attitudes and Predicting Behavior.

[80] Ajzen I., Fishbein M. (1975). A Bayesian analysis of attribution processes. Psychol. Bull..

[81] Allport G. (1935). Handbook of Social Psychology.

[82] Fidan A. (2012). Married Women’s Attitudes toward Intimate Partner Violence against Women in Turkey: An Investigation of Cultural and Structural Explanations.

[83] Triandis H.C. (1971). Attitude and Attitude Change.

